# Energy-resolved neutron imaging and diffraction including grain orientation mapping using event camera technology

**DOI:** 10.1038/s41598-025-96790-1

**Published:** 2025-04-15

**Authors:** Tsviki Y. Hirsh, Andrew F. T. Leong, Adrian S. Losko, Alexander Wolfertz, Daniel J. Savage, Tim T. Jäger, John Rakovan, James J. Wall, Alexander M. Long, Sven C. Vogel

**Affiliations:** 1 Soreq Nuclear Research Center, 81800 Yavne, Israel; 2 Los Alamos National Laboratory, Los Alamos, NM 87545 USA; 3 Forschungs-Neutronenquelle Heinz Maier-Leibnitz, 85748 Garching, Germany; 4 Technical University Darmstadt, 64289 Darmstadt, Germany; 5 New Mexico Bureau of Geology & Mineral Resources, New Mexico Institute of Mining & Technology, Socorro, NM 87801 USA; 6 Nuclear Sector, Electric Power Research Institute, Charlotte, NC 28262 USA; 7 GWW School of Mechanical Engineering, Georgia Institute of Technology, Atlanta, GA 30332 USA

**Keywords:** Neutron imaging, Time-of-flight, Detector development, Imaging techniques, Structure of solids and liquids, Experimental nuclear physics

## Abstract

Time-of-flight neutron diffraction and energy-resolved imaging each provide unique perspectives into material properties. Neutron diffraction is useful for assessing microstructural parameters such as phase composition, texture, and dislocation densities, though it typically provides averaged data over the sampled volume. Energy-resolved imaging, on the other hand, offers both spatial and spectral information by detecting Bragg edges and neutron absorption resonances, which enables detailed mapping of microstructure and isotopic composition. When combined, these techniques have the potential to enrich our understanding of material behavior across different scales, enhancing our understanding of complex materials. Traditionally, these modalities are conducted on separate instruments, which is time-consuming and poses challenges for data integration. Here, we report the integration of the LumaCam, an event-mode energy-resolved neutron imaging camera with the HIPPO time-of-flight diffractometer at LANSCE. This integration enables simultaneous diffraction and imaging across the full spectrum, with analysis optimized for diffraction and Bragg-edge imaging in the thermal range (0.45–10 Å) and resonance imaging in the epithermal range (0.5–3000 eV), facilitating comprehensive multi-modal analysis. We demonstrate its capabilities through case studies, including spatial mapping of grain orientations in a steel sample and accurate thickness estimations for irregular samples including a depleted uranium cylinder and a natural silver-containing mineral specimen. The combined setup enhances real-time sample alignment and provides comprehensive data for crystal structure, texture, and isotopic composition analysis. This approach opens new possibilities for advanced applications in nuclear engineering, archaeology, and materials science.

## Introduction

Neutron imaging and neutron scattering are complementary, non-destructive techniques that can probe materials across a range of length scales. Neutron imaging relies on the attenuation of neutrons passing through a sample to reveal features on the micrometer to centimeter scale^1^. In contrast, neutron diffraction leverages the interaction of neutrons with crystalline structures to investigrate properties across atomistic (e.g., atomic positions, unit cell dimensions), micrometer (e.g., dislocation densities), and macro (e.g., grain orientation distributions/texture, phase fractions) domains^2^. Both techniques are able leverage time-of-flight (ToF) techniques, which exploit the relationship between neutron velocity and energy to enhance the respective measurement. While ToF neutron diffraction has been widely established, recent advancements in hybrid pixel detector technologies, such as *Medipix* and *Timepix* ASICs^3,4^, have enabled significant progress in neutron imaging. The successful integration of these technologies into neutron radiography systems^5,6^ has facilitated the development of ToF-based neutron imaging techniques such as Bragg-edge imaging^7–9^ and neutron resonance imaging^10–12^. These techniques provide unique insights into material structure and composition by analyzing neutron transmission spectra on a pixel-by-pixel basis. While these imaging techniques independently provide valuable material insights, recent studies and subsequent reviews have emphasized the advantages of combining transmission imaging with diffraction measurements, highlighting their complementary strengths for material characterization^13,14^. This multimodal diagnostic technique opens up the ability to characterize samples with varied material compositions, irregular geometries, and internal heterogeneities.

Accurate interpretation of neutron transmission spectra within a given imaging measurement requires a precise understanding of a material’s total neutron cross-section, which is directly influenced by microstructure and environmental conditions. For example, Muhrer et al.^15^ attributed deviations between simulated and experimental thermal neutron cross-sections of lead to sample texture, while Meister et al.^16^ observed variations in the 6.67 eV absorption resonance profile of uranium-238 depending on whether it was in metallic uranium, uranium oxide, or UF_6_ gas. These examples underscore the importance of simultaneous diffraction techniques, which would provide critical information about texture and phase composition, to correctly interpret transmission spectra recorded with imaging. Additionally, at elevated temperatures-essential for reactor operations^17^-combining transmission and diffraction measurements enables direct confirmation of the anticipated crystallographic phase, ensuring the material remains stable and does not decompose during characterization.

Just as adding diffraction capabilities yields benefits to imaging measurements, imaging capabilities can directly enhance diffraction experiments with unique advantages. For instance, imaging can provide critical insights into the spatial homogeneity of hydrogen uptake and release during in-situ diffraction measurements investigation the synthesis or decomposition of hydrogen-bearing materials^18–20^. Additionally, energy-resolved imaging enables the use of Doppler-broadening of neutron absorption resonances to measure sample temperatures without the need for invasive sensors^21^, making it particularly valuable in high-temperature diffraction studies. While this approach has been demonstrated for high-pressure and high-temperature experiments^22^, it remains far from a routine technique as great care needs to be taken in utilizing isotopes with suitable resonances^23^.

In recent years, significant effort has been directed toward establishing dedicated energy-resolved neutron imaging beamlines, often situated adjacent to dedicated neutron time-of-flight diffraction beamlines. Instruments such as ERNI@LANSCE^24,25^, VENUS@SNS^26^, RADEN@J-PARC^27^, ODIN@ESS^28^, and CUPi2D@SNS-STS^29^ represent a developing network of state-of-the-art beamlines designed to push the boundaries of spatially resolved neutron characterization. However, to date, only two facilities, the IMAT instrument at ISIS^30^ and more recently the energy-resolved neutron imaging (ERNI) instrument at CSNS^31^, have routinely offered integrated diffraction and imaging capabilities. Furthermore, recent studies on IMAT probing materials under stress^32^ and generating strain maps in engineering materials^33^ have demonstrated its ability to deliver comprehensive insights into material behavior with simultaneously diffraction and imaging measurements.

HIPPO@LANSCE now joins this suite of instruments in providing combined imaging and diffraction capabilities. The HIPPO (High-Pressure-Preferred Orientation) instrument^34^ is recognized as a well-established time-of-flight (TOF) neutron diffractometer, originally designed for studies of preferred orientation, phase analysis, and texture in polycrystalline materials. Energy-resolved neutron imaging capabilities were integrated into HIPPO with the installation of a state-of-the-art LumaCam system. This system, which employs an event-mode camera coupled to a neutron-sensitive scintillation screen via an image intensifier, offers significant advantages over traditional neutron imaging detectors and other scintillator-based technologies. The LumaCam setup has been successfully utilized for thermal/cold^35^, epithermal^36^, and fast neutron applications^37^. Its versatility in tailoring the scintillator screen and optics for specific experimental needs, along with its compact footprint, makes the LumaCam particularly well-suited for integration with existing neutron diffraction instruments. In comparison to the other combined imaging and diffraction instruments, HIPPO operates under slightly different conditions. Unlike IMAT@ISIS and ERNI@CSNS, HIPPO has a shorter flight path length of roughly 9 meters (compared to IMAT’s 20-meter and CSNS’s 35-meter flight paths) and does not operate curved guides or energy selecting beam choppers. This allows imaging systems on HIPPO to utilize both thermal and epithermal neutrons within a single beam pulse, supporting multiple imaging modalities, such as Bragg-edge imaging and neutron resonance imaging in parallel with diffraction measurements.

This work explores the integration of the LumaCam with the HIPPO instrument for quantitative neutron imaging and diffraction analysis. We demonstrate the combination of Bragg-edge imaging, neutron resonance imaging, and diffraction analysis for materials ranging from geological samples to engineering materials. This synergy unlocks new avenues for characterizing complex material systems, by providing a more holistic view of structural and compositional characteristics, leveraging complementary information from both imaging and spectroscopic measurements.

## Experimental setup

### Facility and beam configuration

**Fig. 1:**
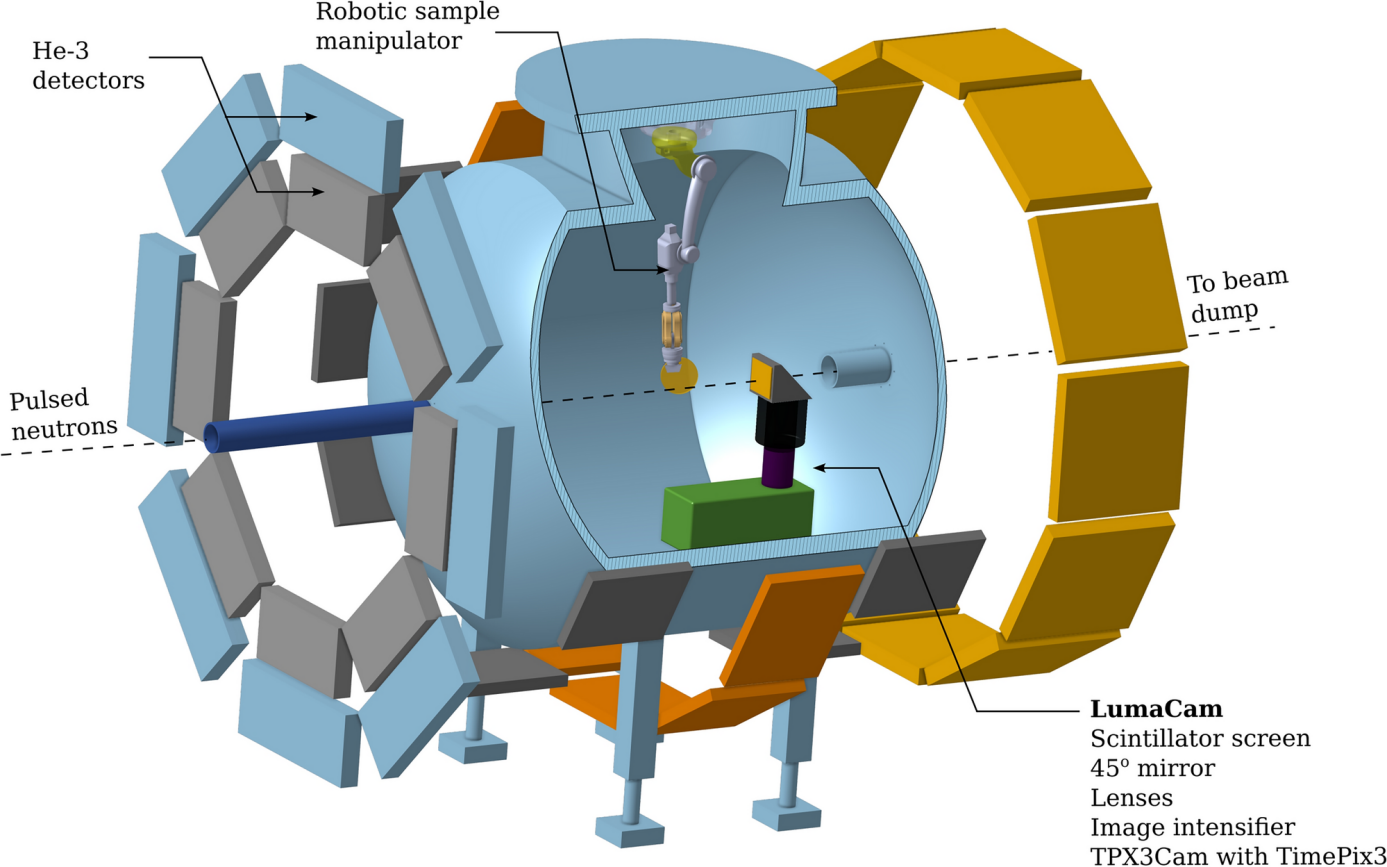
Schematic layout (not to scale) of the HIPPO diffraction instrument on Flight Path 4 at the Lujan Center. The pulsed neutron beam passes through a steel and polyethylene collimator before reaching the imaging system located $\sim 9.0\,{\text{m}}$ from the moderator. Surrounding the HIPPO chamber are 1200 He-3 detector tubes for detecting scattered neutrons. The LumaCam setup is positioned 10 cm behind the sample.

All measurements were carried out at the HIPPO instrument (Fig. 1), located on Flight Path 4 (FP4) at the Lujan Neutron Scattering Center at the Los Alamos Neutron Science Center (LANSCE)^38^. Flight Path 4 views a high intensity water moderator on the lower tier of the Lujan Center’s target-moderator-reflector-shield (TMRS) system^39,40^, which which provides a high neutron flux of $10^{7}\,\hbox {n}/\hbox {s}/\hbox {cm}^{2}$^41^ and is characterized by a peak flux in the thermal energy region with a falling tail extending into the epithermal energy range. The distance between moderator and sample on HIPPO is 8.9 m. The incident beam is collimated by layers of steel and borated polyethylene, resulting in a beam spot size of 10 mm in diameter (14 mm base-to-base) at the sample position. With the collimator entrance aperture of 1 cm and length of 30 cm positioned approximately 130 cm from the sample, the system provides an estimated L/D ratio of ∼ 130. Neutrons scattered off the sample are detected by 1200 He-3 detector tubes mounted on 45 panels arranged on five rings with nominal diffraction angles of 40, 60, 90, 120, and 140 degrees around the main HIPPO sample chamber, covering around 20% of 4π^42,43^. HIPPO is equipped with a robotic sample changer arm^44^ that holds the sample in a prescribed orientation during the measurement and allows for rotations around any selected axis. When coupled with imaging, this functionality enhances the potential for tomographic studies, even for samples with challenging geometries. For the imaging setup, a microscope version of the LumaCam, as described in previous studies^35^, was installed 10 cm downstream from the sample position. The combination of high flux with relatively low background allows for shorter count times in terms of exposures, with white beam radiographs obtained in approximately one minute. In contrast, energy-resolved imaging requires relatively longer acquisition times, as the duration depends on the statistical quality needed for pixel-level or pixel-averaged transmission spectra to perform conclusive analysis. In this work, energy-resolved neutron images were acquired with an average exposure time of anywhere from 10 to 30 minutes per image, which provided sufficient statistical quality for analysis using transmission spectra averaged over pixel regions as low as 16 × 16.

### Neutron imaging setup and specifications

The LumaCam system on HIPPO utilized a ^6^LiF-ZnO:Zn scintillator screen from rcTRITEC^45^, with a thickness of 450 mm and dimensions of 40 × 40 mm. Although ^6^LiF-ZnO:Zn produces approximately half the light output of the more traditionally used ^6^LiF-ZnS:Ag scintillators, it was selected for its superior timing characteristics. Specifically, its primary decay component is significantly faster at 1 μs, compared to the 5 and 80 μs components typical of ZnS-based scintillators^46^, making it better suited for applications requiring superior timing resolution. With each neutron event, scintillation photons are emitted from the scintillator surface and relayed through a lens configuration consisting of a 100 mm lens followed by a 50 mm lens mounted in reverse using a macro-ring adapter. The photons are then directed to a dual-stage image intensifier, which amplifies the incoming light signal by a factor of roughly $10^6$. This amplified optical signal is subsequently projected onto a single 256 × 256 TimePix3 sensor with a pixel pitch of 55 mm. Taking into account the entire series of optical components, the field-of-view at the scintillator imaging plane was around 14 × 14 mm^2^

### Sample materials

This study investigated several samples selected to demonstrate the capabilities of a simultaneous imaging and diffraction setup on HIPPO, ranging from calibration standards to natural and engineered material. For calibration samples, a 200 μm thick tantalum (Ta) foil and a 30 mm thick aluminum can filled with iron powder^47^ were used to calibrate the flight path lengths using well-known Bragg edges and absorption resonances, respectively. In addition to calibration samples, the study examined three additional materials selected for their unique characteristics and relevance to neutron imaging and diffraction studies.A natural silver specimen from the Crow Mine in Lincoln County, NM, was chosen for its irregular, wire-like morphology, making it well-suited for assessing neutron resonance imaging capabilities on complex geometries. A large-grain steel sample, cut from a thick-walled, centrifugally cast austenitic stainless steel light water reactor (LWR) primary cooling loop pipe, was included. The sample, with dimensions of 10 × 12.7 × 80 mm, is ideal for observing Bragg edges due to its potential for relatively large grains within the bulk. Additionally, this sample provided the added benefit of being previously studied and characterized using neutron diffraction, as reported by Schmitt et al.^48^. A depleted uranium cylinder, with dimensions of I5 mm in height and 7 mm in diameter, was included in the study due to its well-known neutron absorption resonances, making it an excellent test case for simultaneous neutron resonance imaging, Bragg-edge imaging, and neutron diffraction.

Here, the beam current was used to integrate up to an arbitrary neutron exposure for each measurement. Given a nominal proton current of 90${\upmu }$A, as reported through an EPICS channel within the LANSCE facility, exposure times were approximately 10 minutes for each sample orientation. For calibration measurements with the iron powder and Ta sheet samples, exposure times were roughly 30 minutes.

### Data acquisition and event reconstruction

The Timepix3 sensor and subsequent SPIDR readout of the LumaCam system provides raw data files that contain detailed information for each pixel hit over a specified threshold, including the x and y coordinates of the pixel position, the time of arrival (TOA) with a resolution of 1.5625 ns, and the time over threshold (TOT) in nanoseconds. Additionally, a $T_{0}$ signal from the initial proton pulse impinging upon the spallation target was recorded via an external time-to-digital converter (TDC) channel on the LumaCam. Data processing was performed using a commercial software package, EMPIR, which employs a two-stage analysis process described in^37^. In the first step, pixel activations are grouped based on a hierarchical single linkage clustering method with a spatial radius and time window. Once pixel cluster group is identified, a photon position and timing was determined by calculating the weighted average of pixel activations within a group, and the timestamp of the earliest pixel activation in the group, respectively. The second step clusters reconstructed scintillation photons using similar spatial and temporal criteria to determine whether scintillation photons originate from a neutron, gamma, or spurious noise. Groups exceeding a predefined threshold of photons are considered neutron interactions. The neutron event’s position is determined as the unweighted average of photon positions within a group, with the earliest scintillation photon arrival time taken as the neutron event time. Non-neutron interactions are filtered out. This algorithm effectively implements pulse shape discrimination for imaging detectors, representing a key advantage of this detector type. Once neutron events are identified, energy information is calculated based on ToF in determining the difference between the $T_{0}$ signal and the neutron interaction timing information.

Based on the field-of-view and the ^6^LiF-ZnO:Zn scintillator used in this study, the parameters used in each stage were as follows: In the first stage, a maximum search radius of 5 pixels and a maximum time window of up to 100 μs were employed to group and reconstruct pixel activations into single photons. During the second stage, the photons were clustered on the basis of at least 2 photons in a cluster within a time window of μs. At this point, photon clusters of a least two or more were classified as a single neutron event, while single-photon events were rejected as likely gamma rays or scintillator afterglow. Additional pulse shape discrimination filtering was applied to photon cluster groups identified as a neutron event to improve data quality where clusters with a time-distribution shorter than 5 μs were rejected as they were most likely gammas. Once neutron events were identified, reconstructed, and filtered, a time-of-flight value was determined as the time elapsed since the last $T_{0}$ from the proton pulse. All final neutron events were binned into stacks of images and saved in the tagged image file format (TIFF). The binning parameters were user-defined based on the imaging modality: for neutron resonance imaging, the binning parameters were X: 512 pixels, Y: 512 pixles, and ToF: 250 ns; for Bragg-edge imaging, the binning parameters were X: 512 pixels, Y: 512 pixels, and ToF: 10 μs.

### Data analysis

To extract quantitative measurements, we modeled the transmission of the neutron image based on the Beer-Lambert law. The transmission, *T*, defined as the ratio of intensity measured with the sample in the beam (*I*) to that of an open beam only ($I_o$), is expressed as:

1 $$\begin{aligned} T = \tfrac{I}{I_o} = e^{-n \sigma '(\lambda ) d}\cdot (1 - B) + B, \quad B = B_0 + B_1 \lambda + B_2 / \lambda ,\quad \sigma '(\lambda ) = R(\lambda ) * \sigma (\lambda ) \end{aligned}$$

Here, $\sigma (\lambda )$ is the total cross-section assumed for a given material, *n* is the atomic weight density in atoms/barn-cm, and *d* is the average sample thickness over the ROI, measured in cm. The background term *B* is modeled as a polynomial function with coefficients $B_0$ (constant background), $B_1$ (linear wavelength dependence), and $B_2$ (inverse wavelength dependence) to account for various instrumental effects. For Bragg edge analysis, we employed the open-source NCRYSTAL package with its python API^49^ to simulate $\sigma (\lambda )$ for polycrystalline materials with and without texture/preferred orientation, while employing the python package LMFIT^50^ to conduct the fits. To account for the broadening of theoretical cross-sections caused by moderation time and detector response, $\sigma (\lambda )$ is convolved with the instrument response function $R(\lambda )$. For Bragg edges, we use the Jorgensen response function^47^, traditionally employed in diffraction studies to describe instrumental broadening effects. For absorption resonances, total cross sections were taken from ENDF-VIII.0^51^. The fitting process was carried out using the well-established open-source SAMMY software^52^, in conjunction with the open-source PLEIADES package^53^, a higher-level Python interface to SAMMY. The instrument response function *R* for resonances follows a functional form developed for the ERNI/FP5 instrument^54^ which views the same moderator as our setup. The parameters of *R* were set to $v_1=6.8$, $v_2=4.3$, $w_1=0.54$, $t_1=5.28~ \upmu {\text{s}} ,$ $T_1=3.48~ \upmu {\text{s}},$ $t_2=2.27~ \upmu {\text{s}},$ and $T_2=5.1~ \upmu {\text{s}}$. These parameters were optimized once to fit our calibration sample measurements and kept constant after calibration. For the resonance analysis in SAMMY, we used the wavelength-dependent background model, available in the software, expressed as $B = B_0 + B_1 /\sqrt{E}+ B_2 \sqrt{E},$ where *E* is the energy in units of eV.

In the following section, we present the results of these analyses, beginning with an assessment of the instrumental background, which plays a crucial role in data quality and interpretation.

## Results

### Background characterization, calibrations, and preliminary studies

Traditionally, the background for epithermal resonance imaging is determined using the “black resonance” method^55^. This approach employs foils with significant absorption resonances at specific energies, effectively absorbing any neutrons at those energies and leaving behind only the background contribution from scattered neutrons. For this study, we used a 200 μm thick Ta foil, which was positioned on the robotic arm at the sample location, 10 cm in front of the event camera.

The resonance transmission spectrum recorded by the event camera for the Ta foil exhibited the typical response expected for Ta, without significant distortions, consistent with recent findings^36^. This spectrum, representing the average transmission across the entire FOV and normalized to the open-beam measurement, was fitted using SAMMY to determine the background contribution.

Analysis revealed that the background remained nearly constant across the 1 eV to 300 eV energy range, contributing only 2.5% to the total transmission. This value is significantly lower than the estimated 20% background or more observed in the neighboring ERNI/FP5 instrument^36^, which has a substantially larger FOV and greater scattering components from the surrounding environment. We also observe a substantial background reduction compared to previous assessments using a different TOF imaging camera on ERNI/FP5^56^. This improvement is primarily attributed to the gamma rejection capability of the event camera.

**Fig. 2:**
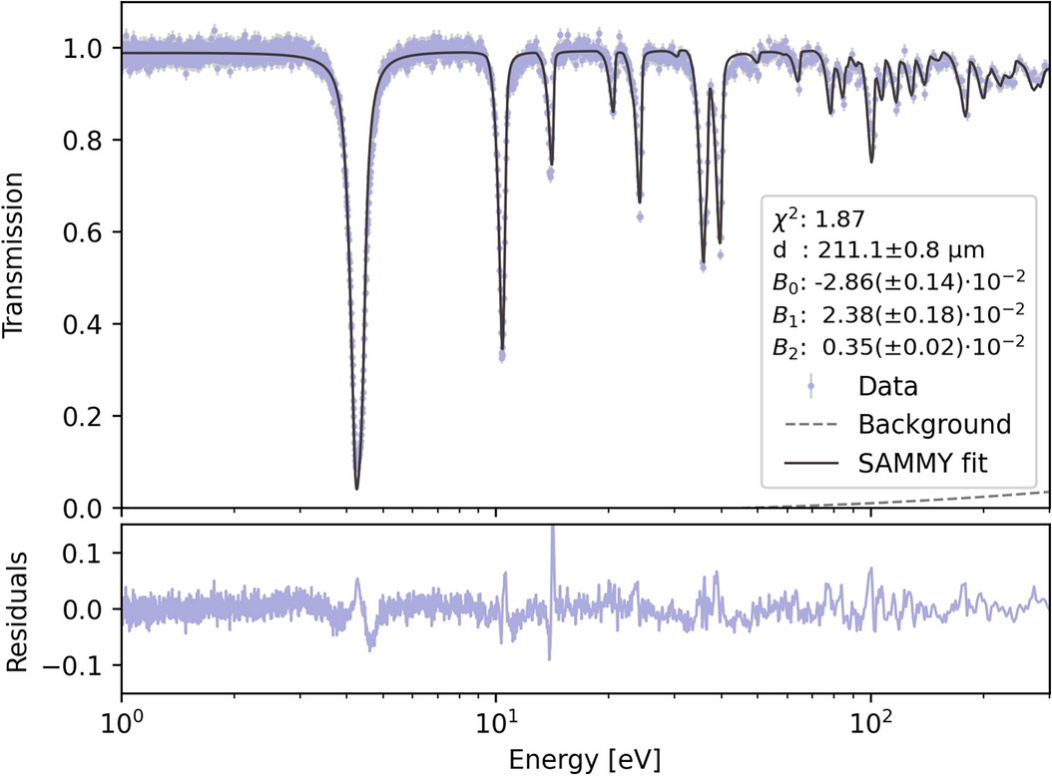
Transmission spectrum for a Ta foil sample. The data (gray circles) exhibit multiple resonance dips characteristic of the Ta isotopes. The SAMMY fit (blue line) accurately models the observed resonances resulting in a sample thickness of *d*=211.1 ± 0.8 μm.

Figure 2 demonstrates the excellent agreement between the measured Ta transmission spectrum and the SAMMY model prediction, highlighting the high quality of data obtained using the HIPPO instrument. The simple transmission model achieved a reduced $\chi ^2$ value of 1.87, indicating a robust fit to the resonance features up to 300 eV. The model predicted a Ta foil thickness of $d=211.1\pm 0.8\, \upmu m$, which is within 5.5% of the measured sample thickness. The discrepancy may be attributed to the shape of the response function, originally measured for FP-5. Future improvements in fit accuracy could be achieved through precise determination of the response function using a high-precision calibration procedure.

We utilized the well-characterized resonance pattern of the Ta foil to calibrate also the TOF parameters, including the flight path distance (9.014 ± 0.001 m) between moderator and event camera and time delay relative to the accelerator trigger signal (0.10 ± 0.01 μs). Given that the nominal moderator-to-sample distance for HIPPO was 8.91 m and the event camera was ${\sim 10}$ cm behind the sample while the time delay between the trigger signal originates from a coil about ${\sim 10}$ m before the 800 MeV protons hit the spallation target, these values are in excellent agreement with the instrumental and facility setup. This TOF calibration, combined with the background estimation, was subsequently applied to the resonance and Bragg edge analysis of the remaining samples in this work.

**Fig. 3:**
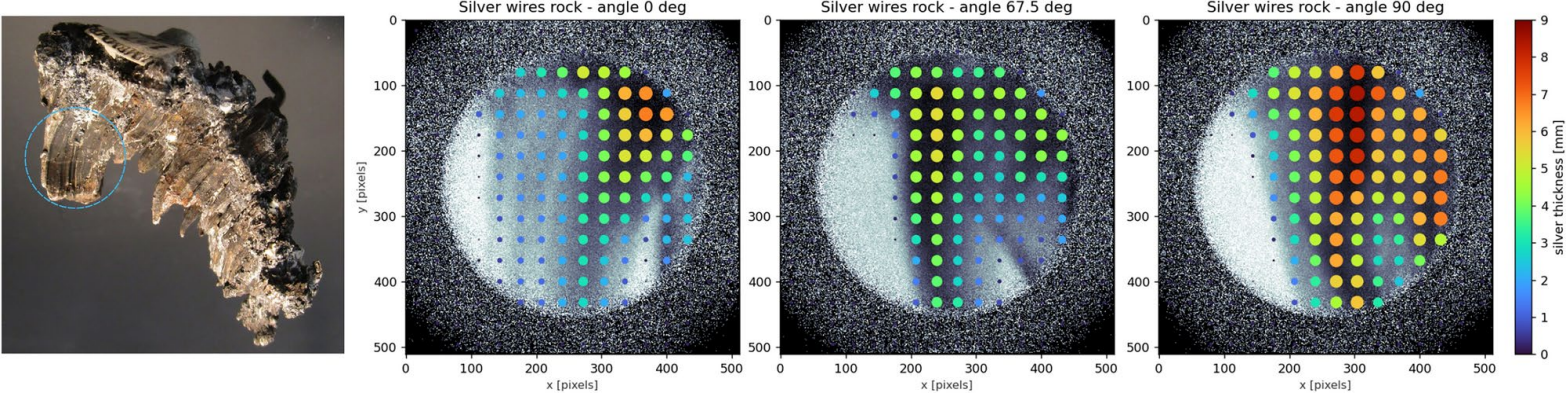
(Left) Natural specimen of silver with a wire-like morphology (New Mexico Bureau of Geology and Mineral Resources Mineral Museum specimen #12674), with the ROI marked by a dashed circle. (Three right panels) Silver thickness maps obtained from individual fits to the neutron transmission data in 32 × 32 pixel subgroups. The marker color and size in each subgroup correspond to the estimated projected silver thickness at orientations of 0°, 67.5°, and 90°. The thermal neutron attenuation is in the background of the scatter plot.

To demonstrate the utility of the epithermal resonance imaging approach, we examined an irregularly shaped wire-silver mineral sample from the Crow Mine, Nogal Mining District, New Mexico. Figure 3, left, shows a photograph of the entire specimen and the ROI (the remainder of the sample was masked by masking tape painted with Gd_2_O_3_ paint during data collection). This sample exhibits a wire-like morphology in the ROI that may result from the growth of native silver via isotope dependent ion conduction of silver through silver sulfide^57^, suggesting a potentially heterogeneous distribution of silver isotopes throughout the sample.

We performed 10 minute count time measurements (at a nominal proton current of 90 μA) with the ROI highlighted in Fig. 3, left, of the sample in the beam at orientations of 0°, 67.5°, and 90° around the vertical axis. For each measurement, we calculated the transmission spectrum across 32 × 32 pixel subgroups (corresponding to a 1.6 × 1.6 mm^2^ area on the sample), balancing the need for adequate counting statistics with spatial resolution given the experimental acquisition time constraints. We then conducted SAMMY model fits with the cross-sections of the two silver isotopes in their natural composition of 51.8 at.% Ag-107, 48.2 at.% Ag-109 to the individual pixel transmission curves, treating the thickness as the only free parameter. For the purpose of this demonstration, we assumed the natural density of silver, 10.49 g/cm^3^, and displayed in Fig. 3 the predicted thickness in each pixel as a color-coded scatter plot, where larger and warmer-colored points indicate locations of higher silver thickness. The background of each plot shows the corresponding thermal neutron image of the sample in their respective orientation.

Figure 3 maps the thickness of the sample, in principle enabling tomographic reconstruction if data for more rotations is collected. This would allow to e.g. identify inclusions within natural mineral specimen or identify different minerals in more complex rock specimen. The additional isotopic density information provided by epithermal resonance imaging aligns well with the actual material distribution observed in the thermal neutron images (background of the scatter plot). Notably, for strong thermal neutron absorbers like silver, epithermal imaging offers a crucial advantage: it provides thickness information that would be difficult to obtain using thermal neutron imaging alone due to the strong absorption of thermal neutrons by elements such as silver, ultimately leading to full attenuation of the thermal neutron beam. In contrast, epithermal neutrons can penetrate deeper into the sample and interact with nuclei at specific resonance energies, allowing for more detailed and quantitative analysis of the silver thickness. With future refinements to our methods and higher statistical precision, we could non-destructively map variations in isotope ratios on the order of a few hundred ppm, similar to those observed by Anderson et al.^57^. This would enable the study of naturally occurring isotope separation phenomena as demonstrated in their work. These findings underscore the value of the epithermal resonance imaging approach in providing quantitative, spatially resolved information about the distribution of specific isotopes within complex, irregularly shaped samples.

Diffraction analysis of the sample, omitted here for brevity, showed the specimen to be polycrystalline and the absence of significant preferred orientation in the region of interest. Weak diffraction peaks of a minor secondary phase were observed in the diffraction data. The identity of this phase is undetermined. However, quantification of the silver texture as well as identification of minor phases, which would not appear in Bragg-edge transmission patterns due to their low phase fraction, low crystal symmetry or due to absorption of thermal neutrons by absorbing materials, underlines the value of combining diffraction with radiographic or tomographic methods for the characterization of materials.

**Fig. 4:**
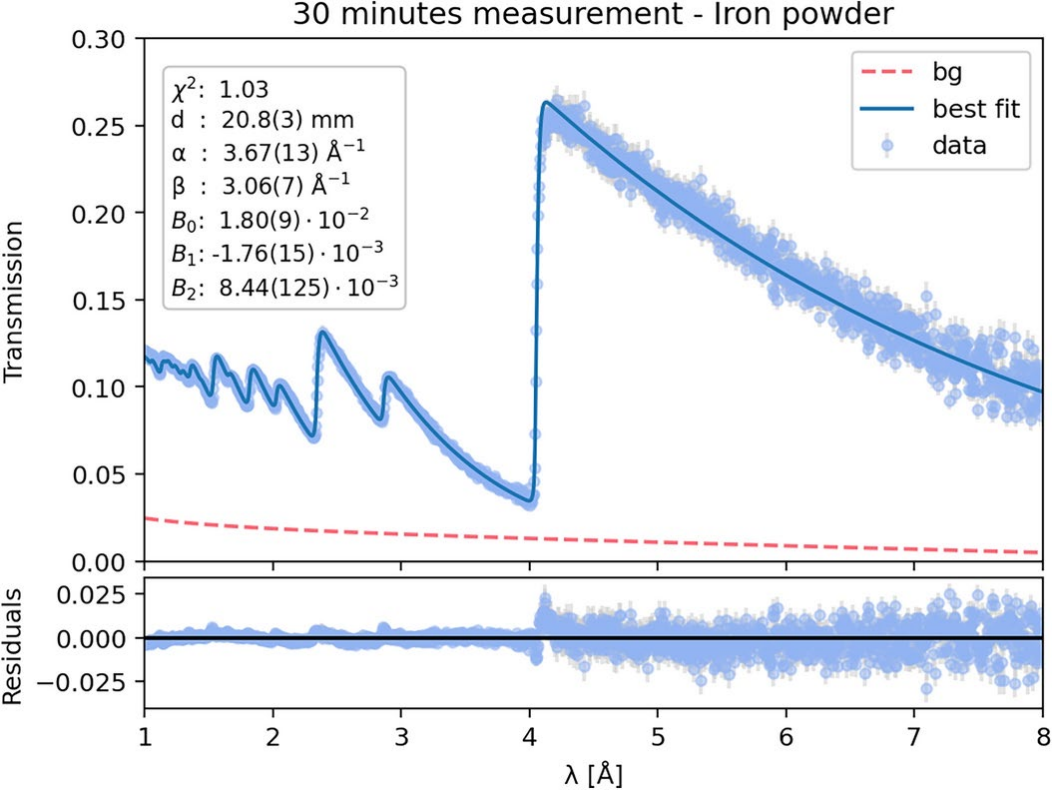
Bragg edge attenuation spectrum of the iron-powder sample. Blue scatter points represent measured data, while the solid blue line shows the NCRYSTAL-based least-squares model prediction. Fit parameters are listed in the legend. The residual plot below the main graph demonstrates that deviations are within 2% of the open beam transmission and centered around zero, indicating an excellent fit quality.

Having established our methodology for absorption resonance analysis, we proceeded to investigate Bragg edges using a standard calibration sample in that of a 30 mm thick rectangular aluminum container filled with homogeneous $\alpha$-phase BCC (Body-Centered Cubic) iron powder^47^. Its uniform composition and well-defined crystallographic properties provide an optimal reference for validating our Bragg edge analysis techniques and assessing instrument performance in this energy range. We utilized the NCRYSTAL package to generate total cross-sections with randomly oriented grains for the $\alpha$-phase iron powder. We then applied the transmission model described in Eq. (1), treating the sample thickness (*d*), flight path length L, time offset and background parameters ($B_0$, $B_1$, $B_2$) as free variables. We set the atomic density (*n*) to $8.49 \times 10^{-2}$ atoms/barn-cm, based on the natural solid iron density of 7.8 g/cm^3^. To account for instrument response, we used the Jorgensen model^47,58^ as our response function for the total cross-section, expressed as $R(\lambda ,\alpha ,\beta ,\sigma ,\Delta )$, with $\lambda$ is the neutron wavelength and $\alpha$, $\beta$, $\sigma$ and $\Delta$ are the model shape parameters. This model is widely employed for diffraction peak shape description in pulsed neutron diffraction, modeling the asymmetric emission of neutrons of a given wavelength from the moderator, on the HIPPO and other neutron TOF diffraction instruments. For this analysis, we allowed the $\alpha$ and $\beta$ parameters to vary, while fixing the Jorgensen model parameters $\sigma$=0 and $\Delta$=1. A least-squares method optimized these parameters, yielding an excellent agreement between the model and experimental data, as shown in Fig. 4. The model predicted a sample thickness of 20.8 ± 0.3 mm, which given the 30 mm actual thickness of the aluminum container, leads to a reasonable 65-70% of theoretical powder packing density. The analysis revealed a low background contribution in the thermal energy regions, again accounting for only 2.5% of the total open-beam spectrum flux. This background estimation and the analytical methods developed here were subsequently applied to Bragg edge analyses of additional samples.

### Texture mapping of large grain steel sample

To demonstrate the capabilities of the enhanced HIPPO instrument on a texture sample, we investigated a large-grained specimen that was a centrifugally cast austenitic stainless steel from a LWR primary cooling loop pipe. This specimen was previously characterized with HIPPO using diffraction, which determined the austenite ($\gamma$) to ferrite ($\alpha$) phase ratio to be approximately 9:1 (see the previous work^48^ for more details on the material and the diffraction characterization). This sample exhibits pronounced texture, comprising regions of few individual austenite and ferrite grains, interspersed with randomly oriented, powder-like regions of these two phases. Solidification texture formed during casting is of interest in primary loop piping since coarse grained austenitic stainless steels are elastically anisotropic, making them difficult to inspect using conventional ultrasonic testing (UT) methods, which is required per ASME Code. A priori knowledge of the texture may facilitate the development of component specific UT methods with corrections for directional ultrasonic velocities, yielding improved signal-to-noise ratios. Mapping the spatial distribution of the individual grain orientations and distinct phases within the microstructure yields critical insights into the material’s properties and processing history, and could be ultilize to provide 3D grain maps for modeling or optimization of processing parameters.

**Fig. 5:**
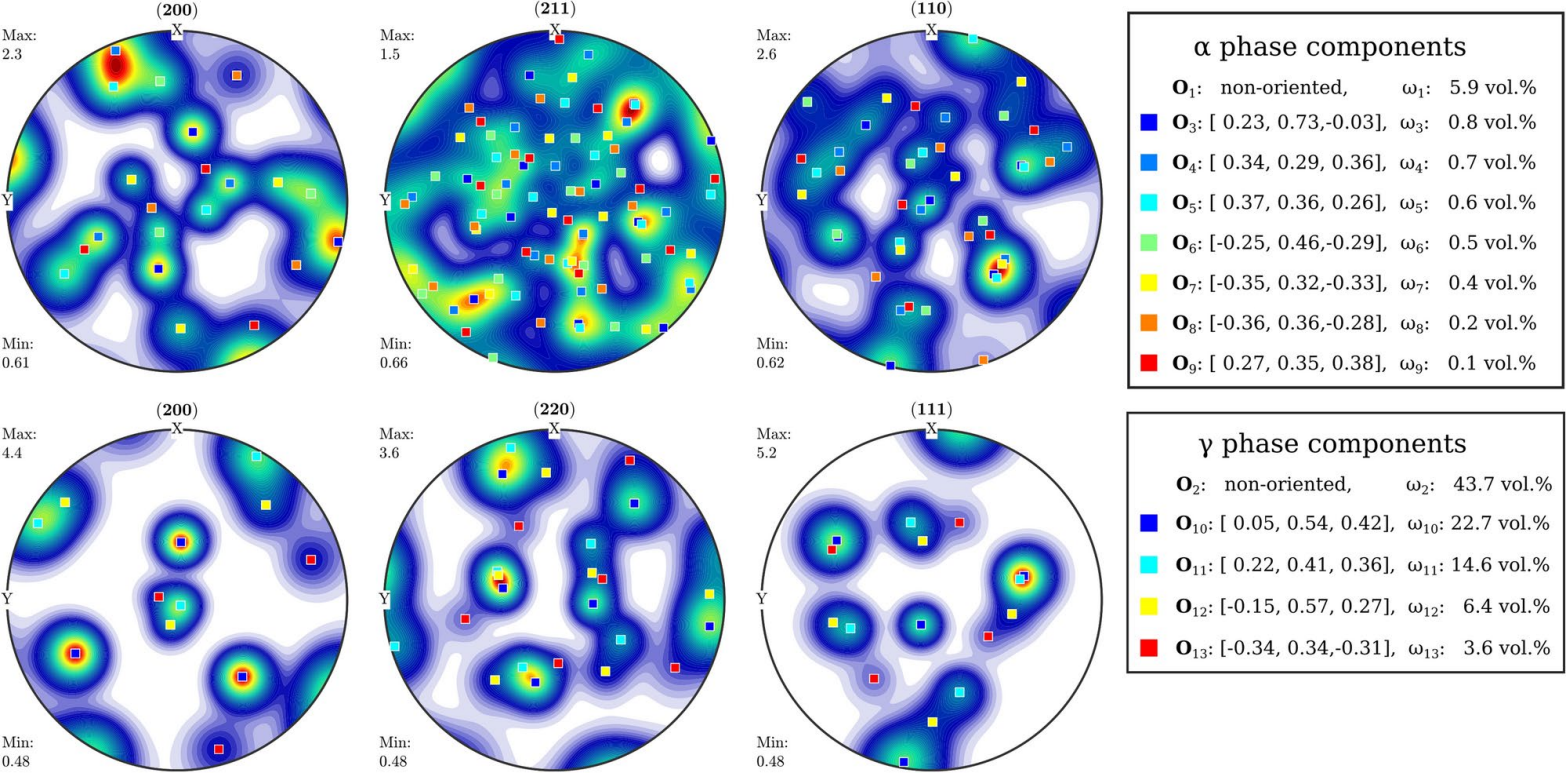
Pole figures for the $\alpha$ and $\gamma$ phases of a large grain steel sample, as determined by texture analysis from the diffraction data. The main orientations in the ZXZ convention and the volume fraction of each component are listed in the legend. These results were used to guide the Bragg edge based texture mapping analysis.

Sample texture, i.e. the orientation distribution function (ODF), is commonly measured with HIPPO using diffraction and the instrument configuration is described in Section "Facility and Beam Configuration". The wavelength-dependent diffraction peak intensities recorded for each scattering angle are fitted using the MAUD software^59^ to generate the ODFs for the sample following procedures described in previous publications^43,60,61^. The ODFs representing the entire volume in the beam (${\sim 1}\,\hbox {cm}^{3}$, different from the ${\sim 2}$ mm slices reported previously^48^) are processed with the MTEX toolbox^62,63^ to determine weight fractions of texture components (orientations) and plot pole figures. Applying this diffraction analysis to our large-grained steel sample, Fig. 5 shows the resultant pole figures for the $\gamma$ and $\alpha$ phases derived from the diffraction analysis, revealing four primary grain orientations for the $\gamma$ phase and 7 for the $\alpha$ phase. Their predicted orientations components, using the ZXZ Euler angles convention^43^, and corresponding average weights are included in the legend. The peak width of each orientation component reflects the OD component broadening of the grains. Both the $\alpha$ and $\gamma$ phases exhibit broadening ranging from approximately 5° to 25°, suggesting a significant degree of subgrain misorientation in some of the primary grain structures. The diffraction analysis also measured the lattice parameter to be 2.865(1) Å for the BCC $\alpha$ phase and 3.5930(1) Å for the FCC $\gamma$ phase. These values are in excellent agreement with 2.8665(2) Å and 3.5974(3) Å reported for pure iron^64^ and a Fe-20.3Cr-11.2Ni (wt%) alloy^65^, close to the composition of the material of this study, respectively.

We supplement the diffraction analysis with energy-resolved Bragg edge imaging by providing spatially resolved reconstruction of the sample’s microstructural features, including individual grain orientations. These orientations are determined from neutron imaging by detecting shifts in the Bragg peak positions and intensity modulations in the transmission spectrum.

Given the large number of possible variables contributing to the transmission spectrum, we used the results of the diffraction analysis to consider only the dominant orientations for each phase. Equation (1) can then be expressed as:

2 $$\begin{aligned} T(\lambda ) = \exp \left( -nd\left[ \omega _1 \sigma _{\alpha }^p(\lambda ,\mathbf {O_1}) + \omega _2 \sigma _{\gamma }^p(\lambda ,\mathbf {O_2}) + \sum _{i=3}^{9}\omega _i \sigma _{\alpha }(\lambda ,\mathbf {O_i}) + \sum _{i=10}^{13}\omega _i \sigma _{\gamma }(\lambda ,\mathbf {O_i}) \right] \right) \cdot \frac{1 - B(\lambda )}{N} + B(\lambda ) \end{aligned}$$

Here, *n* denotes the total atomic number density of stainless steel in atoms/barn-cm, assuming a density of 7.8 g/cm^3^ (neglecting the few percent difference in density for the small amount of ferrite phase), and *d* represents the sample thickness in cm. $\omega _i$ are the relative weights of each orientation $\mathbf {O_i}$. $\sigma ^p$ represents the randomly oriented cross-sections for the $\alpha$ and $\gamma$ phases ($i=1,2$), and $\sigma$ denotes the directional cross-sections for specific orientations $\mathbf {O_i}$ of the $\alpha$ ($3\le i\le 9$) and $\gamma$ ($10\le i\le 13$) phases, as specified in Fig. 5. The weights are constrained to sum to unity. *N* and $B(\lambda )$ represent the normalization factor and wavelength-dependent background function, respectively. To account for the significant amount of scattering in this specific sample, the background was modeled with an additional parameter proportional to $\lambda ^2$. The parameters *d*, $\omega _i$, *N*, and *B* were fit to the transmission data for each ROI using LMFIT.

In this study, we interpret the mosaicity parameter in NCRYSTAL as a broadening factor for a texture component. This is analogous to the FWHM broadening parameter used in the “Standard Functions” ODF description in MAUD, or the corresponding texture component broadening parameters in MTEX. Neutron diffraction data alone cannot distinguish between a single, several millimeter-sized grain with misorientation from an average orientation or a group of potentially spatially separated smaller grains with a similar orientation distribution. Although Bragg-edge imaging has the potential to address this issue, the current resolution of our dataset is insufficient for further investigation. Previous metallographic studies of this sample have confirmed the presence of millimeter-sized grains in this type of material. Each orientation was assigned a fixed mosaicity value $\eta _i$ between 5° and 25°, predetermined through calibration process to ensure convergence. Our calibration analysis indicated that the model is more sensitive to the relative weights $\omega _i$ of each orientation component than to the $\eta _i$ values.

**Fig. 6:**
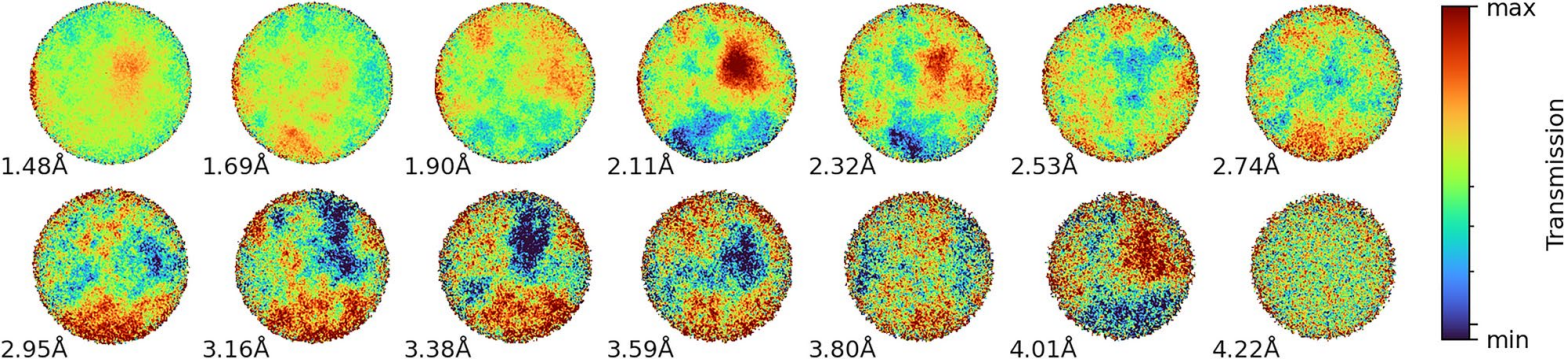
Wavelength-averaged neutron transmission images of a large grain steel sample. Each panel represents a different wavelength bin, with the lower bound of the bin indicated. The color scale represents relative neutron intensity, with red (hot) and blue (cold) indicating higher and lower transmission, respectively. The spatial variations in intensity reveal the distribution of differently oriented grains and their wavelength-dependent impact on neutron transmission.

The LumaCam recorded wavelength-resolved spectra at each pixel, generating 512 × 512 pixel frames at 10 μs time intervals for each of the three projections. Figure 6 presents the sequence of wavelength-averaged transmission images, with each panel labeled by the lower bound of its corresponding wavelength bin. Observed spatial variations in intensity illustrate the substantial impact of different grain orientations on the observed neutron transmission. Regions of high and low intensity, visualized as “hot” and “cold” areas, respectively, indicate localized variations in neutron attenuation. Areas corresponding to large grains, each with a single crystal orientation, are clearly discernible as extended clusters of high or low intensity. These intensity variations represent enhanced or reduced neutron transmission at specific wavelengths. It is important to note that these transmission images represent the areal, through-thickness average of grain orientations along the neutron path through the sample.

To validate our methodology, we initially analyzed the average transmission through the sample using a ROI encompassing the entire beam spot. While this approach lacks spatial resolution, it should yield orientation weight predictions that closely correspond to the volume-averaged results from diffraction texture analysis. The measured transmission was fitted using the aforementioned model, with $\omega _i$, $B_j$ and *N* as varied parameters while *d* was kept fixed at 12.7 mm for the $0^\circ$ projection and 10 mm for the $90^\circ$ projection, and $\eta _i$ values were assigned based on the mosaicity estimates from the pole figures.

**Fig. 7:**
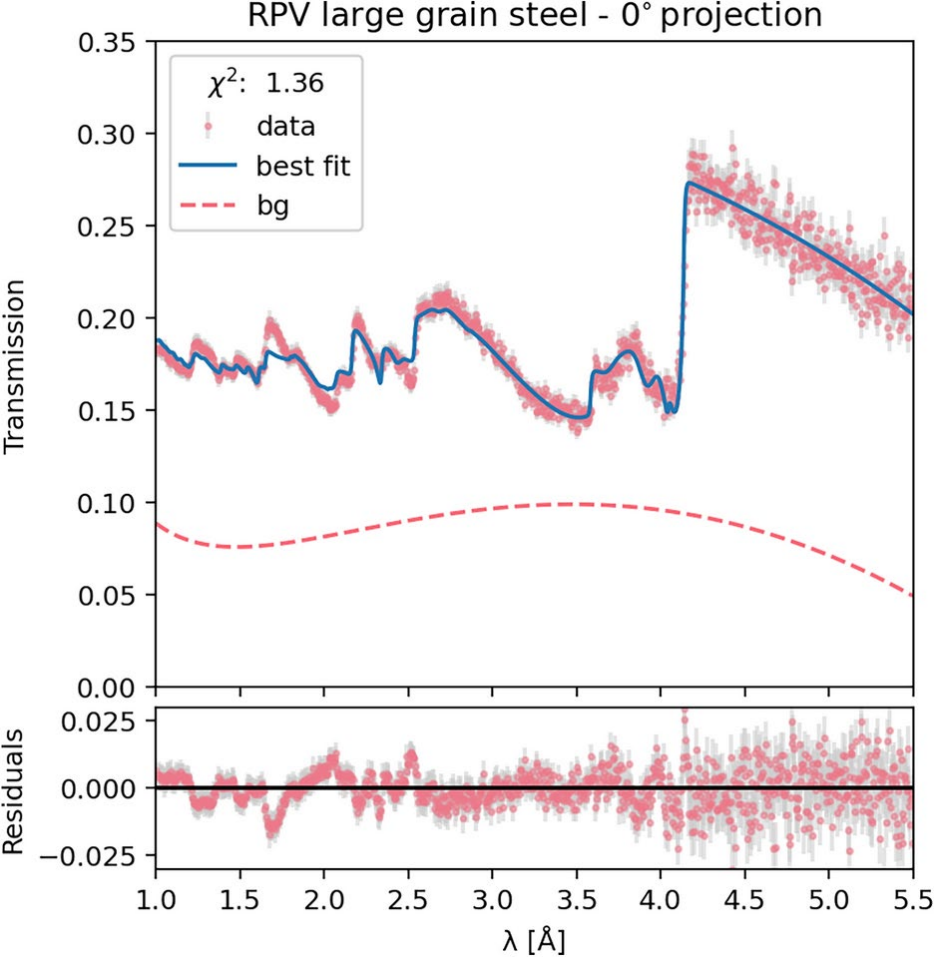
Average transmission spectrum and corresponding best-fit results for the full ROI of the 0° projection of the steel sample. The upper panel shows the measured data (red points), the overall fit (blue line), and the background estimation (dashed pink line). The lower panel displays the fit residuals.

Figure 7 presents the results of our analysis for the 0° sample orientation. The upper panel displays the transmission data alongside the best-fit prediction. The fit closely reproduces the distinctive features of the data across a broad wavelength range, with the exception of a few under-fitted features, resulting in a reduced $\chi ^2$ of 1.36, indicating good fit quality. The estimated background, which remains below 10% across the relevant wavelength range, is also shown. The predicted background is substantially higher than that found for the iron-powder sample, possibly due to the higher density and increased neutron scattering of the steel sample. Notably, the predicted orientation weights for the $\gamma$ phases, which constitute the majority of the grains in the sample, align with the diffraction-estimated values within 30%. This agreement is satisfactory, considering that the two methods measure projection versus volume averages, which are not necessarily identical.

**Fig. 8:**
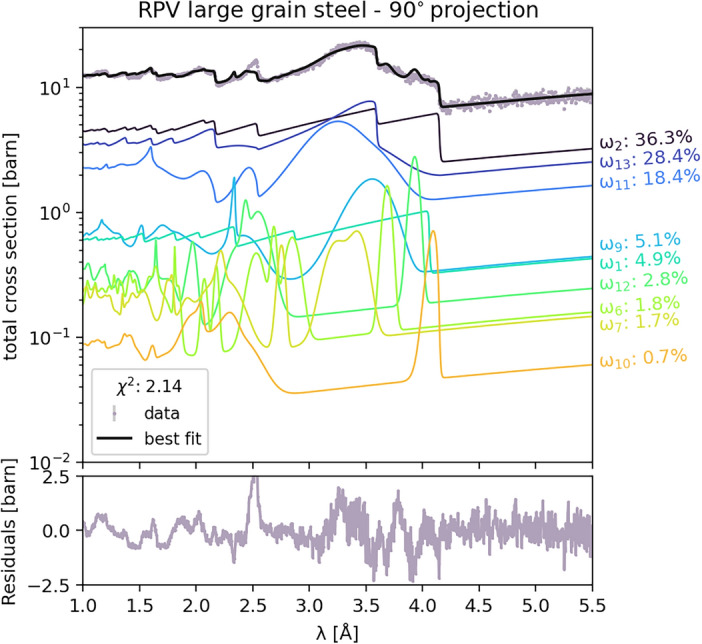
Fit to the 90° projection of the steel sample, showing the total cross section and contributions from different orientation components. The upper panel displays the measured data (gray points), the best fit (black line), and individual component contributions (colored lines). The weight percentage of each component is indicated next to its corresponding curve. Randomly oriented $\alpha$ and $\gamma$ phases are $\omega _1$ and $\omega _2$, while $\omega _{3--9}$ and $\omega _{10--13}$ represent their specific orientations. The lower panel shows the fit residuals.

Figure 8 shows the fit results of the 90° projection, converted to the equivalent total cross-section, allowing for the breakdown of the relative contributions of the different orientation components. The dominant $\omega _i$ weights are indicated next to each component curve, demonstrating the contribution of each component to the overall shape of the cross-section and the model’s fit to the data. The bottom panel presents the residuals in units of barns, showing an acceptable overall match. Despite the complex and distinctive features in the dataset, the fit residuals demonstrate consistent agreement across the entire relevant wavelength range.

**Fig. 9:**
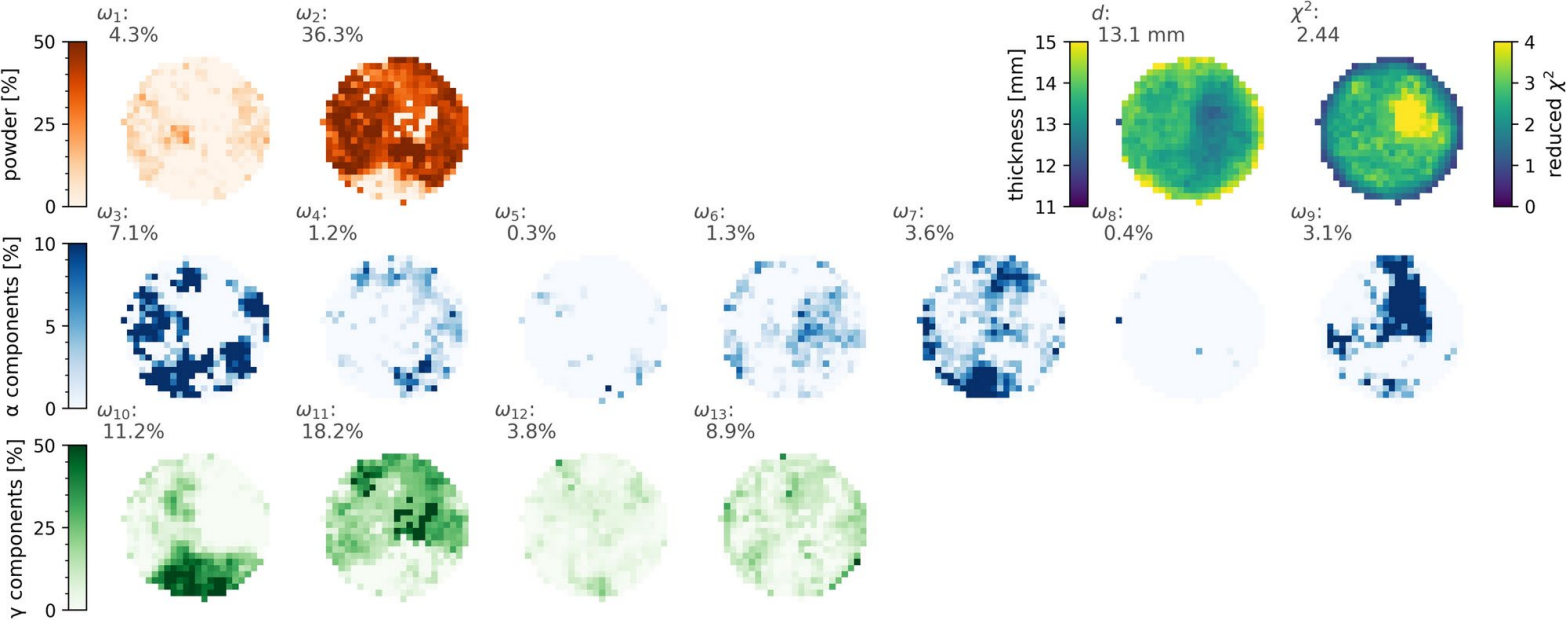
Spatial mapping of texture components derived from best-fit analyses across 16 × 16 pixel subgroups. The figure presents 13 orientation components with their average weights shown above each panel: two for $\alpha$ and $\gamma$ powder phases ($\omega _1$–$\omega _2$, top row in red, 0–50%), seven for $\alpha$ phases ($\omega _3$–$\omega _9$, middle rows in blue, 0–10%), and four for $\gamma$ phases ($\omega _{10}$–$\omega _{13}$, bottom row in green, 0–50%). Color intensity indicates the relative contribution of each orientation component, with scales shown on the left for each group. The maps demonstrate the concentration of grains with specific orientations in distinct regions of the sample. The upper right panels display the thickness map (d) in mm (average 13.1 mm) and the reduced-$\chi ^2$ map (average 2.44), indicating the goodness of fit across the analyzed area.

Next, we divided the 0° projection image into 16 × 16 pixel ROIs to enable spatial mapping of the grain orientations. This binning was chosen as a practical compromise to balance statistical accuracy with noise reduction, given the limitations of acquisition time. The background, normalization, and mosaicity parameters were fixed according to the values determined from the full ROI analysis of that projection, while $\omega _i$ for all 13 powder and grain orientation phases were varied. The resulting $\omega _i$ predictions for the 13 components were compiled into spatial density maps and displayed in Fig. 9, showcasing the relative contributions of different orientation components across the sample, along with the thickness distribution and goodness of fit.

Figure 9 reveals a complex grain structure with distinct regions dominated by specific orientations. The $\gamma$ phase exhibits large, well-defined grains: $\omega _{10}$ shows a substantial grain concentrated at the bottom of the beam spot, while $\omega _{11}$ and $\omega _{13}$ display prominent grains in other areas. These $\gamma$ orientations effectively fill the gaps in the powder component $\omega _2$, suggesting that large $\gamma$ grains are embedded in a random matrix comprising approximately half the sample volume. The $\alpha$ phase orientations display a similar morphology, although some components exhibit more dispersed grains. Notably, the local distributions of grains with orientations $\omega _9$ and $\omega _3$ complement each other, forming a “negative” of one another: regions with a high concentration of $\omega _9$ grains correspond to areas where $\omega _3$ grains are absent, and vice versa. This complementary pattern is also observed between $\omega _7$ and $\omega _3$. Interestingly, the powder components ($\omega _1$ and $\omega _2$) contribute significantly to the overall texture, with average weights of 4.3% for $\alpha$ and 36.3% for $\gamma$ phases, respectively, as labeled in the figure. These values closely align with our earlier validation using the average transmission analysis, which in turn corresponded well with the predicted randomly oriented weights from diffraction analysis (5.9% for $\alpha$ and 43.7% for $\gamma$). The other orientation components ($\omega _3$ through $\omega _{13}$) also demonstrate good agreement with the predictions from diffraction texture analysis. For instance, the $\alpha$ phase components ($\omega _3$–$\omega _9$) have individual weights ranging from 0.3% to 7.1%, while the $\gamma$ phase components ($\omega _{10}$–$\omega _{13}$) range from 3.8% to 18.2%. These values are consistent with the overall texture distribution expected from our diffraction results. The $\chi ^2$ values show a decrease at the border of the circular region due to lower statistics, while they are elevated in a specific area near the center. This increase in $\chi ^2$ likely reflects the model’s incomplete representation of the orientation components in that central region. It is worth noting that this analysis is based on a single projection from a ten-minute acquisition. Extending the acquisition time would enable the use of smaller ROIs, potentially yielding finer spatial resolution. Furthermore, this approach opens up possibilities for tomographic scans or analysis approaches combining the three sample orientations typically measured for HIPPO texture analysis, both could facilitate the desired three-dimensional texture reconstruction using similar methodologies.

**Fig. 10:**
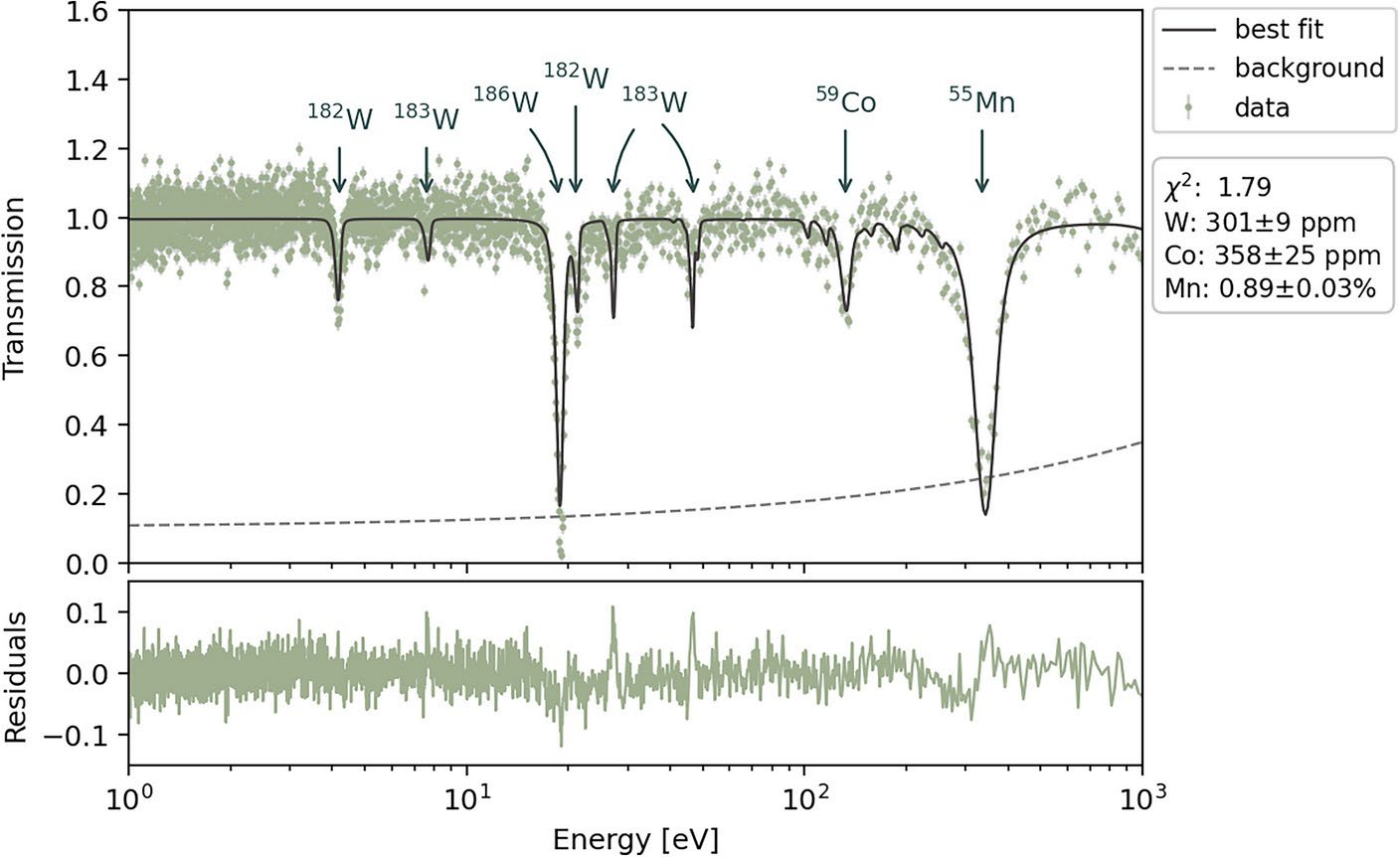
Transmission spectrum of the large-grain steel sample in the epithermal energy region up to 500 eV. The experimental data (brown points) exhibit resonance features attributed to 0.89% Mn, 358 ppm Co, and 301 ppm W impurities. Notably, only Mn and Co were listed in the original material specifications. The SAMMY fit (solid black line) accurately models the observed resonances, including those from the previously unidentified W impurity. The dashed line represents the estimated background. Lower panel shows the residuals between the fit and the data.

Finally, we investigated the steel sample’s isotopic composition using absorption resonance patterns, where an unexpected feature emerged. This sample, being a section of a LWR primary loop pipe, was presumed to have a well-characterized composition. According to Schmitt et al.^48^, the specified composition is (Fe-Bal) Cr(20.69%) Ni(9.57%) Mn(0.89%) Mo(0.14%) Si(1.10%) Cu(0.1%) Co(0.03%) V(0.04%) P(0.023%) C(220 ppm) S(30 ppm) O(53 ppm) N(572 ppm). Within this energy range, only cobalt and manganese were expected to exhibit significant resonances. Surprisingly, Fig. 10 shows additional resonance structures not listed by Schmitt et al. Based on the peak positions and their amplitude, we identified these as belonging to tungsten (W) contaminants. To quantify the relative compositions of the resonance-exhibiting isotopes, we fitted the transmission spectra using the SAMMY code. Assuming the Mn contribution aligns with the 0.89% specification, our fit indicates 358 ± 25 ppm of Co, which agrees with that reported by Schmitt et al., and 301 ± 9 ppm of the W contaminant, not part of the previously reported elemental analysis. The presence of tungsten was also not reported in previous characterizations of the sample material^66^. We hypothesize that its presence may be attributed to arc melting of the steel ingot prior to casting. Further investigation may be necessary to determine the origin of this contamination and its potential effects on the material’s properties and performance under operational conditions.

This example highlights the benefits of combining diffraction characterization with Bragg edge imaging and other imaging techniques as well as neutron absorption resonance analysis for a more complete material characterization than diffraction or neutron imaging alone can provide. Even with only ten minutes of count time on the HIPPO instrument, this example demonstrates that such analysis can provide crucial information about material composition and purity, microstructure, and phase composition, all of which could impact material behavior and reliability.

### Analysis of bragg edges and resonances in a depleted uranium sample

**Fig. 11:**
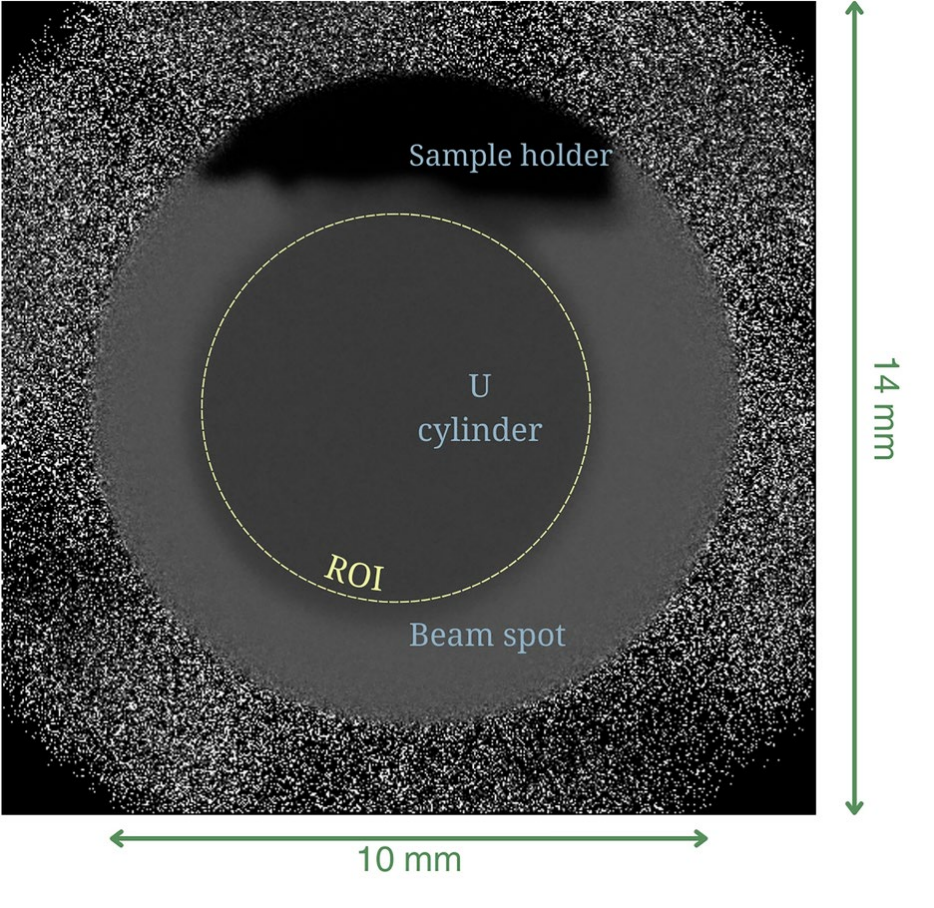
Thermal neutron radiograph of a 5 mm thick depleted uranium cylinder resulting from ten minutes count times. The dashed circle indicates the ROI used for analysis. The beam spot is 10 mm in diameter, and the FOV is 14 × 14 mm^2^.

We demonstrate the synergistic utility of diffraction and imaging techniques on a 5 mm thick depleted uranium metal cylinder in the orthorhombic $\alpha$-U phase exhibiting both resonances in the epithermal region and diffraction patterns and therefore Bragg edges in the thermal energy region. Measurements were recorded at 0°, 67.5°, and 90° over a ten minutes integration time per sample rotation. Figure 11 illustrates the thermal neutron radiograph of the sample, with the ROI used for this analysis outlined by a dashed circle.

**Fig. 12:**
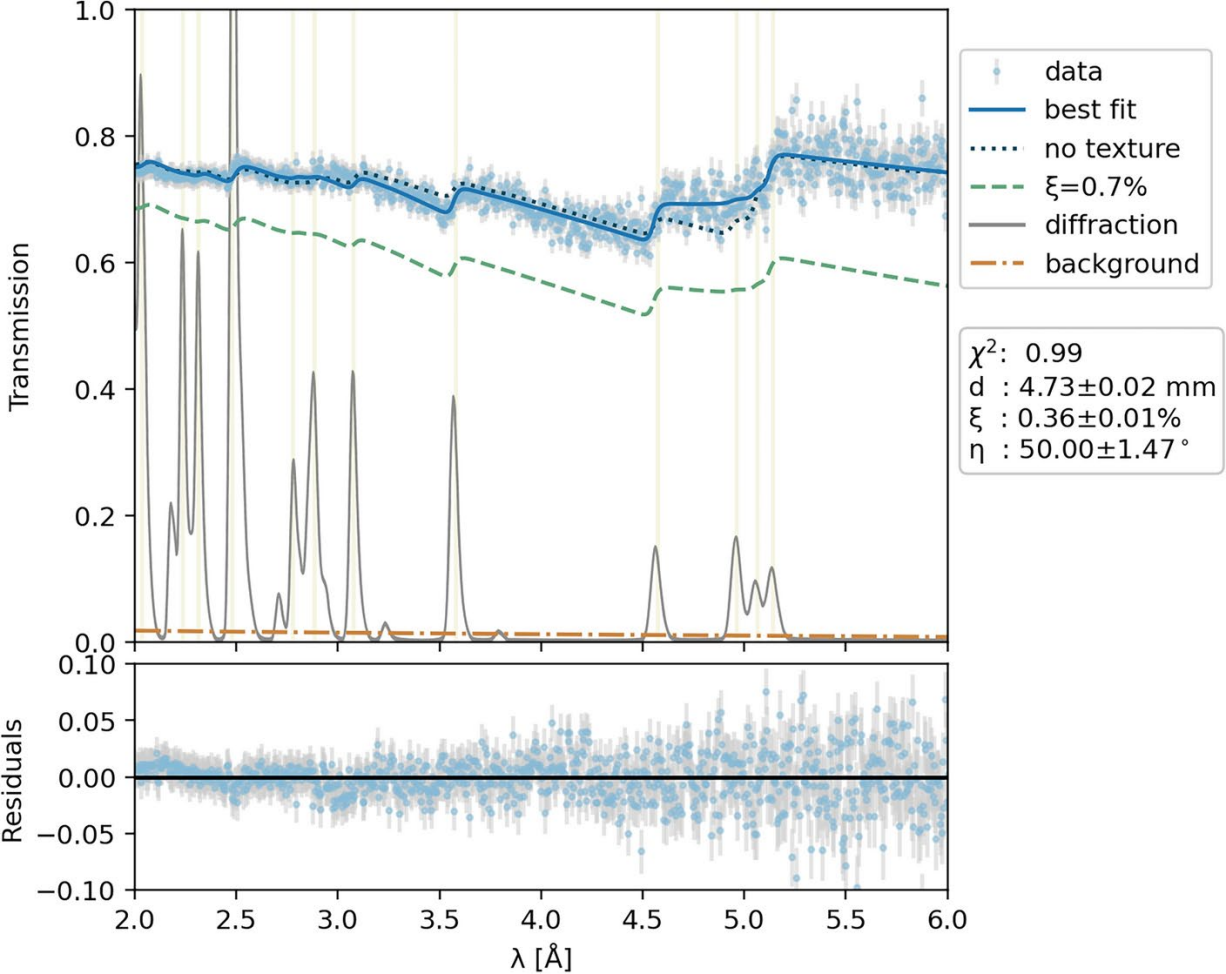
Average measured Bragg edge transmission through the ROI (light blue points) with the fitted transmission model (solid blue line). The diffraction pattern (grey line) is overlaid, with its x-axis corresponding to $\lambda = 2d$ instead of the usual d-spacing. The lower panel shows the fit residuals. The dotted line represents transmission assuming no texture. The dashed green line shows transmission for natural uranium enrichment ($\xi =0.7\%$). Note the correlation between the positions and intensities/heights of diffraction peaks and Bragg edges.

**Fig. 13:**
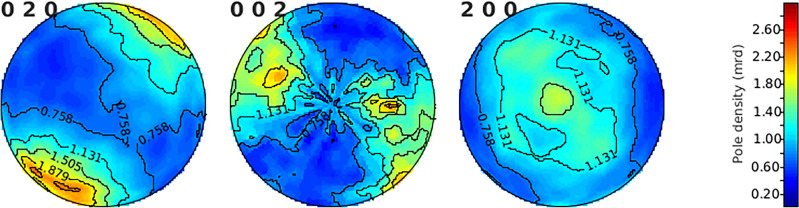
Pole figures for the $\alpha$-U cylinder sample, as determined by texture analysis from neutron diffraction data.

Diffraction analysis performed using HIPPO confirmed the sample to be purely orthorhombic $\alpha$-phase uranium. The neutron diffraction bulk texture analysis determined that the ODF of this sample can be described by a single texture component with Euler angles of (0°, 25°, 2.5°) relative to the beam axis with a broadening of approximately 50° as illustrated in the pole figures in Fig. 13. This deformation texture is consistent with a history of rolling and cutting from larger stock material. The diffraction pattern is included as an overlay to Fig. 12 along with the Bragg edge pattern recorded for that sample in the sample orientation with the cylinder axis parallel to the incident neutron beam. The x-axis associated with the diffraction pattern corresponds to $\lambda =2d$ rather than the usual d-spacing, measured with the 90° detectors. Notably, the positions and intensities of the diffraction peaks align well with the positions and heights of the Bragg edges, providing qualitative confirmation of the expected relationship between these two complementary measurement techniques, and the benefit of performing diffraction and imaging simultaneously on the same instrument.

For the Bragg edge analysis, the oriented cross-section for $\alpha$-uranium metal obtained from the diffraction analysis was simulated using NCRYSTAL^67^. However, that data pertains to naturally depleted uranium (nominal 0.7% U-235), while our sample exhibited less absorption due to deeper depletion and lower U-235 concentrations. To address this discrepancy, we included an additional parameter, $\xi$, representing the enrichment percentage contributed by the additional absorption cross-section $\sigma _{\text {abs}}$ of U-235. The scattering cross-section in U-235 is negligible compared to the absorption cross-section, justifying the exclusive addition of the absorption component in our model:

3 $$\begin{aligned} T(\lambda ) = \exp \left( -nd\left[ \sigma _{\text {tot}}(\lambda ,\textbf{o}_{hkl},\eta ) + (\tfrac{\xi }{0.7\%}-1) \cdot \sigma _{\text {abs}}(\lambda ) \right] \right) \cdot \frac{1 - B(\lambda )}{N} + B(\lambda ) \end{aligned}$$

Here, $\xi$ becomes zero when the enrichment equals the natural enrichment of 0.7% U-235, already represented by the total cross-section term. Deviations from this value predict different enrichment levels. $\sigma _{\text {tot}}$ is convoluted with the response function $R(\lambda )$, based on the Jorgensen model, as calibrated for the iron powder sample. We fixed $B(\lambda )$, $R(\lambda )$, and *N* to the values determined during the calibration process using the iron powder sample. During fitting, we varied thickness *d*, mosaicity $\eta$, and enrichment percentage parameter $\xi$.

Figure 12 shows good agreement between the recorded and fitted transmission data, yielding an excellent $\chi ^2$ value close to unity and small residual values shown in the lower panel. Sample thickness estimated from the fit is 4.73 ± 0.02 mm. The predicted enrichment level is $\xi$=0.36 ± 0.01%, indicating deeper depletion than natural uranium, which is typical for American-produced depleted uranium. The green-dashed line in the plot illustrates the expected transmission assuming natural uranium enrichment. The significantly higher observed transmission underscores the sensitivity of thermal neutrons to U-235 content. Measuring the entire energy-resolved Bragg edge spectrum offers greater precision compared to traditional white beam or monoenergetic neutron measurements for estimating the sample thickness. This advantage stems from the richer information obtained by fitting the complete Bragg edge spectrum, enabling precise decomposition of the absorption cross-section contribution relative to incoherent and coherent scattering, the latter of which can vary significantly with texture. The fit strongly favored a high mosaicity texture component of $\eta = 50 \pm 1.5^\circ$, which represents the upper limit of this parameter calculable in NCRYSTAL. The dotted line in Fig. 12 represents the prediction assuming no texture, and the difference between the models is significant beyond uncertainty. The texture of the sample as illustrated by pole figures in Fig. 13 is substantial, which in combination with the anisotropy of the thermal expansion of $\alpha$ uranium^68^ would lead to a strong distortion of the cylindrical sample shape during temperature changes. Repeated thermal cycling, inducing fully reversible thermal expansion in most materials, will lead for $\alpha$-uranium with similar textures to permanent changes of the sample geometry, so-called thermal ratcheting^69^. It is therefore of paramount importance to control the texture of $\alpha$-uranium components. However, the differences in the Bragg edge transmission patterns for the cross-sections with and without preferred orientation in Fig. 12 are not very pronounced. This may indicate that texture analysis from Bragg edge data is generally difficult and in particular for lower crystal symmetry systems such as $\alpha$-uranium.

Due to the short ten-minute measurement, insufficient counting statistics were obtained to detect spatial changes in lattice parameters or texture. A higher-statistics measurement could facilitate better interrogation of the crystalline structure of uranium-containing samples using this imaging technique, potentially allowing mapping of different phases and online identification of interesting regions for diffraction inspection with scattered neutrons. To our knowledge, this represents the first measurement of its kind for uranium Bragg edges. Even with low counting statistics, we demonstrate the ability to measure sample thickness, enrichment level, and texture with good precision using a simple model. A measurement of higher statistical quality may provide analysis of crystalline structure, mapping of residual strain, phase and texture in uranium samples, online identification of interesting regions for diffraction inspection of scattered neutrons, with potential relevance to nuclear industry applications.

**Fig. 14:**
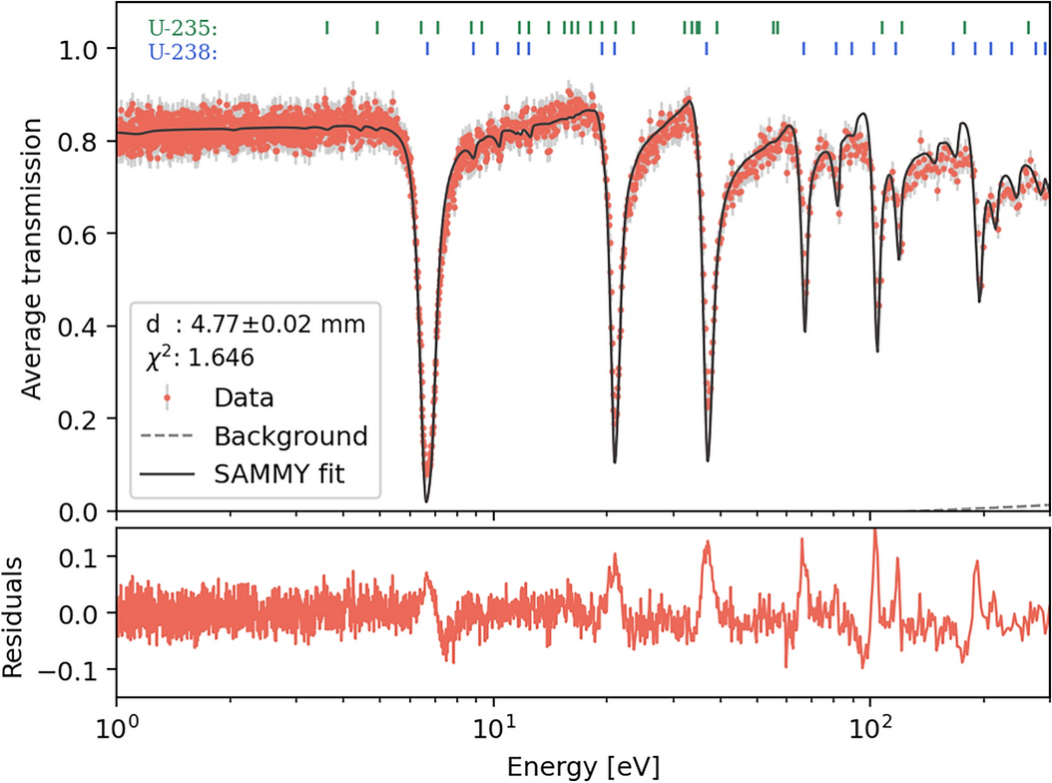
SAMMY fit of the neutron transmission data for a 5 mm thick depleted uranium cylinder. The plot shows good agreement between the measured data (red circles) and the calculated SAMMY fit (dark solid line). The residuals are plotted in the lower panel. The sample thickness *d* was the only parameter varied, yielding a value of 4.77 ± 0.02 mm, within statistical consistency, with the prediction from our Bragg edge analysis. The top of the figure indicates the positions of major resonances for U-238 (blue) and U-235 (green).

Absorption resonance analysis of the uranium sample was also performed over the same ROI shown in Fig.  11. The recorded TOF data was binned into 250 ns intervals and converted to energy for fitting a transmission model using SAMMY, following an approach similar to that used for the tantalum sample. We applied the background parameters estimated from the tantalum sample analysis, varying only the sample thickness parameter *d* and assuming a depleted uranium metal density of 18.95 g/cm^3^^70^. In Fig.  14, the sample thickness is calculated to be 4.77 ± 0.02 mm, which is in agreement with the 4.73 ± 0.02 mm thickness estimated from our Bragg edge analysis. The consistency between these two independent methods enhances our confidence in the results and suggests that the sample may have a lower density than anticipated for depleted uranium.

To verify this finding, we conducted post-analysis measurements. The sample mass was determined to be 3.765(1) g, with a thickness of 5.03(1) mm and a diameter of 7.24(4) mm. These measurements yield a density of ${18.18(20)}\,\hbox {g}/\hbox {cm}^{3}$, approximately 4% lower than the expected value for depleted uranium. We observed no surface flaking or UO_2_ diffraction peaks, which would have indicated oxidation of the sample, resulting in mass change and slight volume expansion. Using this updated density value to recalculate our thickness predictions, we obtain 4.97(6) mm from Bragg edge analysis and 4.93(6) mm from resonance analysis, in consistency with the actual measured thickness.

The absence of resonances associated with uranium isotopes other than U-238, and the wavelength-dependent absorption modeling during our Bragg edge analysis, strongly indicates that the sample has indeed been depleted of U-235 to low levels. The SAMMY model incorporated a 0.36% U-235 content, as predicted by the Bragg edge analysis. The locations of the main resonance energies for both isotopes are indicated at the top of the plot. None of the U-235 resonances are discernible above the statistical noise, precluding a precise determination of the enrichment level from this measurement alone. This rapid ten-minute measurement demonstrates the level of precision achievable with this method, highlighting its potential value for future analyses of actinide-bearing samples requiring accurate isotopic and microstructural characterization as required by nuclear applications of such materials.

**Fig. 15:**
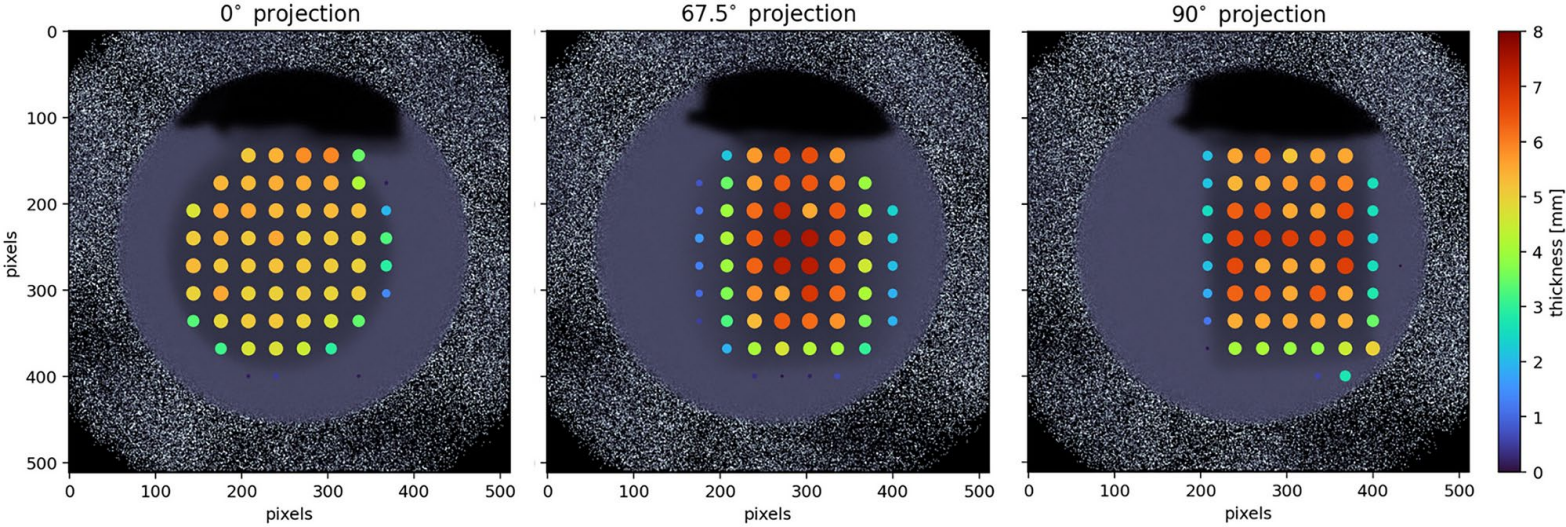
Isotopic density maps for U-238, obtained from individual SAMMY fits to 32 × 32 subgroups of pixels in the neutron images for the three sample orientations. Markers are overlaid on each projection orientation, with color and size representing the fitted projection thickness for each pixel subgroup.

Besides characterization of the bulk sample based on data from the entire field of view, we also performed fits to the transmission spectra from the resonance neutron energy region of of 32 × 32 pixel ROIs, as described above in section 3.1. Only the thickness *d* was varied. Figure 15 overlays the thickness map with the associated thermal neutron image for the three different sample rotations. The average thickness calculated in each ROI matches the known projected thickness with an accuracy estimated to be better than 20%. A longer count time of the sample would allow for pixel-by-pixel reconstruction of the thickness and isotopic content, potentially enabling tomographic reconstruction of irregularly shaped samples of interest. The combination with a diffraction instrument would facilitate focusing on interesting parts of the sample and investigating them simultaneously without the need to remove the sample from the experimental stage.

## Summary and outlook

This study demonstrates the advantages of integrating a neutron-sensitive LumaCam event camera with a diffraction instrument at HIPPO, enabling simultaneous diffraction and imaging across both thermal and epithermal energy ranges. Our results showcase how this combined approach provides complementary information to quantify complex, irregularly shaped samples with high precision and time efficiency.

We calibrated the system using well-characterized Ta and Fe-powder samples, demonstrating efficient gamma background suppression with background levels below 2.5%. In analyzing a large grain steel sample, we spatially mapped the ferrite and austenite phases using Bragg edge imaging, complementing quantitative texture analysis from diffraction data with spatial information where the texture components are located. For a depleted uranium sample, we achieved thickness estimations with <2% accuracy, consistent within statistical uncertainty, using both thermal (Bragg edge analysis) and epithermal (resonance analysis) neutrons, in just 10 minutes of acquisition time. We also accurately predicted projection thicknesses for irregular samples, including a depleted uranium cylinder viewed in three different rotations (with the cylinder axis perpendicular to the beam) and a natural silver mineral specimen. Additionally, we demonstrated spatial mapping of silver isotope densities in a sample that is highly absorbing for thermal neutrons.

The integration of the LumaCam with HIPPO offers several key benefits. It provides real-time sample alignment and positioning through live neutron imaging feedback, and enables complementary analysis of crystal structure and texture via diffraction and Bragg Edge measurements as well as isotopic composition via nuclear resonances. This approach also facilitates rapid data acquisition and analysis, even for complex, irregularly shaped samples, and enhances capabilities for studying highly neutron-absorbing materials.

Looking forward, this combined diffraction-imaging approach opens up exciting possibilities for future applications. In nuclear power engineering, particularly in fuel development, the ability to measure Bragg edges of uranium compounds (oxides, nitrides, carbides, alloys etc.) enables the mapping of residual strain and phase identification in nuclear fuels, including pre- and post-irradiation testing. This technique also holds significant potential for non-destructive analysis in archaeology, cultural heritage, and geology, offering detailed insights into the structure, composition, and isotopic distribution of ancient artifacts and mineral samples. In additive manufacturing, this approach enhances quality control by allowing simultaneous mapping of internal strains and crystal orientations through Bragg edges and diffraction measurements. Combining imaging and diffraction methods also optimizes the efficiency with which neutron beams are used since a characterization requiring in most cases two instruments can be done in a single beam time. This approach is particularly advantageous for hazardous samples, as it minimizes necessary sample movements. Diffraction data collected during tomography scans yield superior statistical quality, facilitating the identification of minor phase fractions below the detection limit of shorter, individual measurements. For *in situ* studies, sensor-less sample temperature measurements from resonance Doppler broadening offer unique advantages to improve data quality with more reliable temperature measurements while also offering multi-probe studies of solid state physics phenomena^71^.

In conclusion, the integration of event-based neutron imaging with diffraction at HIPPO represents a significant advancement in neutron scattering instrumentation. This approach provides researchers with a powerful tool for comprehensive material characterization, offering new avenues for discoveries in materials science, engineering, and beyond. The continued development and application of this technology promise to unlock further insights and innovations across various fields, from nuclear engineering to cultural heritage preservation.

## Data Availability

The datasets generated and/or analyzed during the current study are not publicly available due to their size, but are available from the corresponding author on reasonable request.
